# Experimental Study on the Effects of Curcumin in Alleviating Oxidative Stress and Inducing Differentiation in Neural Stem Cells

**DOI:** 10.1155/ijfo/7647912

**Published:** 2026-09-20

**Authors:** Chunyang Wu, Jingfeng Ouyang, Jiaojiao Cheng, Bo Bian, Ziqi Zhang

**Affiliations:** ^1^ Experimental Research Center, Beijing Key Laboratory of Research of Chinese Medicine on Prevention and Treatment for Major Diseases, China Academy of Chinese Medical Sciences, Beijing, China, cacms.ac.cn; ^2^ Dongzhimen Hospital, Beijing University of Chinese Medicine, Beijing, China, bucm.edu.cn; ^3^ Traditional Chinese Medical College, North China University of Science and Technology, Tangshan, Hebei, China, ncst.edu.cn

**Keywords:** curcumin, differentiation, neural stem cells, oxidative stress, Parkinson′s disease

## Abstract

Parkinson′s disease, a neurodegenerative disease that poses a serious threat to human health, is showing a worrying rise in incidence with the global trend of population aging. Among the many treatment options, stem cell therapy undoubtedly brings new hope for this persistent disease. Notably, curcumin (Cur) significantly protected C17.2 neural stem cells (NSCs), primarily by enhancing their resistance to oxidative stress. Based on these findings, the present study delved into the effects of Cur on NSCs, with a special focus on its mechanism of action in oxidative stress alleviation and cell differentiation promotion. The experimental results showed that under oxidative stress conditions, Cur‐treated C17.2 cells not only exhibited enhanced survivability but also their reactive oxygen species (ROS) levels were significantly suppressed, whereas antioxidant indexes showed significant improvement. Remarkably, Cur also demonstrated the ability to promote the transdifferentiation of C17.2 cells to neurons and astrocytes, a process that was accompanied by a significant activation of the Wnt3*α*/*β*‐catenin/Cyclin D1 signaling pathway. This finding provides new ideas for understanding the mechanism of Cur′s role in the regulation of neuroplasticity and stem cell repair and reveals that the Wnt3*α* signaling pathway may become a key target for the treatment of Parkinson′s disease.

## 1. Introduction

Against the backdrop of an increasingly aging demographic structure in contemporary society, Parkinson′s disease (PD), a persistent neurological disease characterized by limb tremor [1], is spreading at an alarming rate in the elderly population [2]. In‐depth investigation of the pathological mechanisms of this complex disease, it is not difficult to find that the progressive degeneration and death of dopaminergic (DA) neurons in the substantia nigra region of the brain constitute its core pathological basis. It is worth noting that the development of neurodegenerative diseases is often closely related to oxidative stress, and this view has reached a broad consensus in the medical community [3]. When the delicate balance between oxidative and antioxidant systems within the body is disrupted, reactive oxygen molecules will increase exponentially, triggering a series of chain reactions. This physiological disturbance will eventually lead to cellular dysfunction and even irreversible cell death, thus accelerating the disease process [4].

Recent years have seen growing interest in stem cell therapy for PD treatment. However, ROS‐mediated oxidative stress environments limit the survival, proliferation, and differentiation of neural stem cells (NSCs) [5]. Within the vast array of consumable goods, certain natural edibles double as therapeutic agents—referred to as Food–Medicine Homology (FMH)—occupying a special niche in nutritional practices. These dual‐purpose substances bridge the gap between sustenance and healing, playing a distinctive role in how we approach nourishment [6]. Curcuma longa L., a perennial herb of the genus *Curcuma* in the family Zingiberaceae, is listed in both *Chinese Pharmacopeia* and *the List of Items That Are Both Foods and Medicines*, serving as a typical medicinal and edible resource. Curcumin (Cur), a natural polyphenol extracted from turmeric rhizomes, exhibits multifaceted biological effects by interacting with various molecular and cellular targets. Modern pharmacological studies have demonstrated that Cur exerts anti‐inflammatory, antioxidant, antitumor, and lipid metabolism‐regulating activities, showing potential application value in the prevention and treatment of chronic diseases. Studies have found that in terms of antioxidation of neurodegenerative diseases, Cur can play a neuroprotective effect on rotenone (Rot)‐induced PD rat model by inhibiting oxidative stress, activating antioxidant system, regulating neurotransmitter imbalance and the expression levels of tyrosine hydroxylase and dopa decarboxylase [7].

Before investigation shows that Cur, a natural compound, exhibits unique biological effects in antioxidant and anti‐inflammatory properties in NSCs, plays a key role in the protective mechanism of NSCs, which provides a new idea for the therapeutic strategy of PD [8].

The Wnt/*β*‐catenin signaling pathway serves as a crucial regulator of cellular proliferation, differentiation, and stem cell maintenance, with its dysregulation frequently implicated in a range of diseases, including neurodegenerative disorders. Within the brain, this pathway operates at various stages of the neurogenic lineage. Specifically, activation of Wnt/*β*‐catenin signaling directs the neuronal differentiation of proliferating NSCs and facilitates, in a dose‐dependent manner, the activation and differentiation of quiescent NSCs. The canonical Wnt pathway is integral to embryogenesis and the formation of neurogenic niches, playing a critical role in the proliferation and differentiation of neural progenitors [9]. Enhanced Wnt/*β*‐catenin signaling promotes the differentiation of neural stem/progenitor cells into neuroblasts. Given these fundamental roles, this pathway emerges as a promising target for understanding and treating PD, where augmenting endogenous neurogenesis could provide therapeutic advantages [10].

It is valuable to conduct an in‐depth investigation into the potential of Cur to modulate oxidative stress levels through a complex network of molecular mechanisms, such as the Wnt/*β*‐catenin signaling pathway, while preserving cellular homeostasis during the differentiation of NSCs. This exploration could elucidate the intricate mechanisms underlying nervous system repair.

## 2. Materials and Methods

### 2.1. Cell Culture

The C17.2 cells were procured from Dalian Meilun Biotech Company (PWE‐MU030). The growth medium used for cell proliferation is a DMEM base, fortified with 10% fetal bovine serum (FBS), 2 mML‐glutamine and a 1% penicillin‐streptomycin mixture (100 U/mL and 100 *μ*g/mL, respectively), both acquired from Gibco in the United States. For differentiation purposes, the cells are maintained in a DMEM‐based medium. They thrive in a controlled environment within a humidified incubator, kept at a balmy 37°C and a CO_2_ concentration of 5%. Upon achieving 90% cell coverage, the cultures are treated with a 0.25% trypsin solution (also from Gibco, United States) to facilitate subculturing.

### 2.2. Identification of C17.2 NSC

The C17.2 cells were planted in 24‐well plates, each equipped with a 14‐mm diameter coverslip coated with poly‐L‐lysine. Following a 48‐h incubation in growth‐promoting culture medium, the cells were tagged with Nestin‐specific antibodies (Proteintech, China) to pinpoint the NSCs. Visualization and recording of the fluorescent labeling were carried out using a state‐of‐the‐art digital slide scanner (Olympus, Japan). Thereafter, the growth medium was swapped out for a differentiation medium, and the transition of C17.2 cells into specialized types was tracked under the microscope a day later.

### 2.3. Evaluation and Determination of Cur Agent Dosage

In exploring the safe application of Cur (Sigma, United States), C17.2 cells were inoculated in a 96‐well plate, and the tolerance of the cells to different concentrations of Cur was analyzed through a comparison between the control group and the treatment group. Cur was administered at concentrations of 1, 2, 4, 8, 12, 16, 32, 64, and 100 *μ*mol/L, showing the correlation between drug concentration and cell activity in an all‐round way. After 24 h of incubation, introduced the CCK‐8 assay (Meilun, China) to gain insight into the changes in cell metabolic activity. During the experiment, the mixed CCK‐8 solution with DMEM culture medium at a ratio of 1:9, and dispensed it each well under the premise of setting a blank control. After incubation at 37°C for 60 min, the absorbance data were captured at 450 nm with the help of Synergy H1/H1MFD microplate reader (BioTek, United States).

### 2.4. Establishment of PD Model

In order to ascertain the precise concentration of Rot (Sigma, United States) necessary to create a PD model, C17.2 cells were planted into 96‐well plates and allocated into two distinct groups [11]: a control batch and several batches treated with Rot. These cells were subjected to an array of Rot concentrations, specifically 0.01, 0.02, 0.04, 0.08, 0.16, 0.32, 0.64, 1.25, 5.00, and 10.00 *μ*mol/L. Following a 24‐h exposure and cultivation phase, the vitality of the cells was gauged via the CCK‐8 assay, enabling the computation of the IC50 threshold.

### 2.5. Protective Effect of Cur on C17.2 Cells

To verify and extend the previous findings, we used the same cell model and Cur concentration (2.0 *μ*mol/L) as in reference [8] to independently repeat the antioxidant assays (ROS, GSH, GPx, and SOD) in Section 2.6 and 2.7, with no data shared from that study.

The cells were planted into 96‐well plates and divided into four distinct categories: control, model, a Cur‐treated cohort, and a positive control cohort. Within the Cur‐treated cohorts, a spectrum of five different concentrations of Cur was examined, specifically 0.5, 1.0, 2.0, 4.0, and 6.0 *μ*mol/L. The positive control cohort received three doses of resveratrol (Res), which were 2.5, 5, and 10 *μ*mol/L, respectively. Both the Cur‐treated and positive control groups underwent a 2‐h pretreatment with their designated compounds before the addition of Rot to every group except for the control, at a terminating concentration of 0.32 *μ*mol/L. Post a 24‐h incubation, the initial culture medium was removed, and the cells′ viability was gauged via the CCK8 assay.

### 2.6. ROS Content Detection

Six experimental groups were constructed to investigate the effects of Cur on cellular oxidative stress. In addition to the control, model, three gradient concentrations of Cur‐treated groups named Cur‐1, Cur‐2, and Cur‐4 (1.0, 2.0, and 4.0 *μ*mol/L), and a positive control group represented by Res (5.0 *μ*mol/L) were included. Before the introduction of the oxidative damage factor Rot (0.32 *μ*mol/L), the cells in the experimental groups were subjected to a 2‐h drug pretreatment phase. After 24 h of incubation, the cells underwent a double wash with a serum‐free medium before being labeled with the DCFH‐DA fluorescent indicator. The cells were incubated at 37°C for 30 min, and the fluorescence signal at 488 nm was captured with the help of electric intelligent microscope (ECHO, United States). The fluorescence intensity was quantitatively analyzed by ImageJ analysis platform, which finally revealed the change pattern of ROS levels in each experimental group.

### 2.7. Restore System Indicator Detection

Glutathione (GSH), glutathione peroxidase (GPx) activity, and total superoxide dismutase (SOD) activity were the three key parameters of interest in investigating the recovery of the antioxidant capacity of the C17.2 cells after Cur treatment.

The cells were inoculated in 60 mm petri dishes and treated in groups according to the protocol in 2.6. The cells were lysed using 300 *μ*L of phosphate‐buffered saline (PBS) (Meilun, China) per 10^6^ cells, followed by a centrifugation step for 10 min at specific conditions (4°C, 10,000 × g). The resultant supernatant was siphoned off and utilized for quantifying GSH levels via a GSH colorimetric assay kit (Elabscience, China).

In order to investigate the activity of GPx, cells were mixed with Western and IP lysis buffer (Beyotime, China) at a ratio of 100 *μ*L per 10^6^ cells and lysed, followed by a centrifugation step for 10 min at specific conditions (4°C, 12,000 × g). Subsequently, the resultant supernatants were employed for assessing GPx activity through a total GPx assay kit (NADPH method) (Beyotime, China).

To quantify the total SOD activity, the cells were disrupted using 100 *μ*L of the SOD extraction buffer from a total SOD activity assay kit (WST‐8 method) (Beyotime, China). Post incubation, the mixture was subjected to centrifugation at 4°C and 12,000 rpm for 5 min. The resultant supernatant was then employed to ascertain the total SOD activity.

Protein levels were quantified using a high‐sensitivity BCA protein assay kit (Beyotime, China), with absorbance readings taken at 562 nm. From these measurements, the GSH content, GPx activity, and total SOD activity per sample unit were subsequently derived.

### 2.8. Western Blot (WB) Analysis and Immunofluorescence (IF) of C17.2 Cell Differentiation

In order to investigate the effect of Cur on the differentiation process of C17.2 cells under oxidative stress, the experimental design selected a concentration of 2 *μ*mol/L. In the experiment, cells were inoculated into 100 mm culture dishes and divided into three independent experimental units, namely, control, Model, and Cur. To ensure accurate normalization of protein loading, *β*‐actin was used as the housekeeping control. For each of the three independent experimental units (control, model, and Cur), the *β*‐actin loading control was run separately in corresponding individual lanes. Following 24 h of growth in complete medium to allow for cell adherence, the culture medium for all groups was replaced with differentiation basal medium (DMEM supplemented with 0% FBS, 2% N2 supplement, 1% penicillin‐streptomycin, and 2 mML‐glutamine). Cells in the Cur were pretreated with Cur for 2 h. Subsequently, except for the control, all groups were exposed to Rot at a final concentration of 0.32 *μ*mol/L. In terms of differentiation assessment criteria, the researchers regarded cells with neural protrusions longer than twice the diameter of the cell body as the basis for determining successful differentiation.

RIPA lysate buffer (Beyotime, China) was used to extract cellular proteins, followed by WB to analyze the differentiation process. Several protein indicators were selected: neuron‐specific nuclear protein (NeuN) (Abcam UnitedStates), glial fibrillary acidic protein (GFAP) (Proteintech, China), and 2′,3′‐cycloadenylate‐3′‐phosphodiesterase (CNPase) (Proteintech, China) as the evaluation criteria for the degree of differentiation. Additionaly, NeuN IF staining was used.

In the protein processing session, the quantification and normalization process was executed by mixing the samples with the loading buffer (Beyotime, China) and denaturing them in a metal bath at 100°C for 5 min. Subsequent experimental steps included SDS‐PAGE electrophoretic separation and PVDF membrane transfer. The membranes were treated with a one‐step WB kit (CWBIO, China) for blocking and primary antibody incubation. Finally, the target proteins were detected by an ultrasensitive ECL chemiluminescence kit (Proteintech, China) and 680RGB high‐sensitivity chemiluminescence imaging system (GE Healthcare, United States), and the data were analyzed by quantitative grayscale analysis using ImageJ.

### 2.9. WB Analysis of the Differentiation Mechanism of C17.2 Cells

Cells were cultivated and collected using the same methods as detailed in Section 2.8. WB analysis was carried out to look into the signaling pathways that promote the differentiation of C17.2 cells. For signaling pathway studies, the experiments focused on the changes in the expression levels of Wnt3*α*, GSK3*β*, *β*‐catenin, and Cyclin D1 (Abclonal, China).

### 2.10. Statistic Analysis

The experimental data processing was performed using GraphPad Prism 9.1. All data were expressed as $\bar{x}\pm s$. For intergroup difference analysis, the independent samples *t*‐test was employed, whereas comparisons among multiple groups were conducted using one‐way analysis of variance (ANOVA). The significance level for statistical testing was set at *p* < 0.05.

## 3. Results

### 3.1. C17.2 Identification of NSC

**Figure 1:**
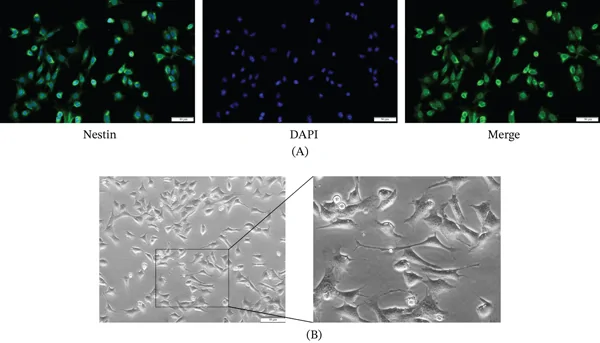
Exploring the properties of C17.2 NSCs. (A) C17.2 cells were nurtured in a growth‐promoting medium for 2 days, after which they were subjected to immunofluorescence labeling using Nestin (highlighted in green), a telltale sign of NSCs identity. Magnification: 200×. (B) Cells were first allowed to proliferate for 1 day before being shifted to a differentiation medium for a further 24 h period. The capacity of the C17.2 NSCs to generate synapses was assessed. Right section: 200× magnification.

Cellular IF staining demonstrated that nearly all C17.2 NSCs in the in vitro culture system expressed Nestin protein (Figure 1A). After 24 h of induced differentiation, synaptic formation became evident (Figure 1B). These findings suggest that the C17.2 cells were in an undifferentiated state and possessed the potential for differentiation, making them suitable for subsequent experimental procedures.

### 3.2. Cur Protects C17.2 NSC From Oxidative Stress‐Induced Damage

**Figure 2:**
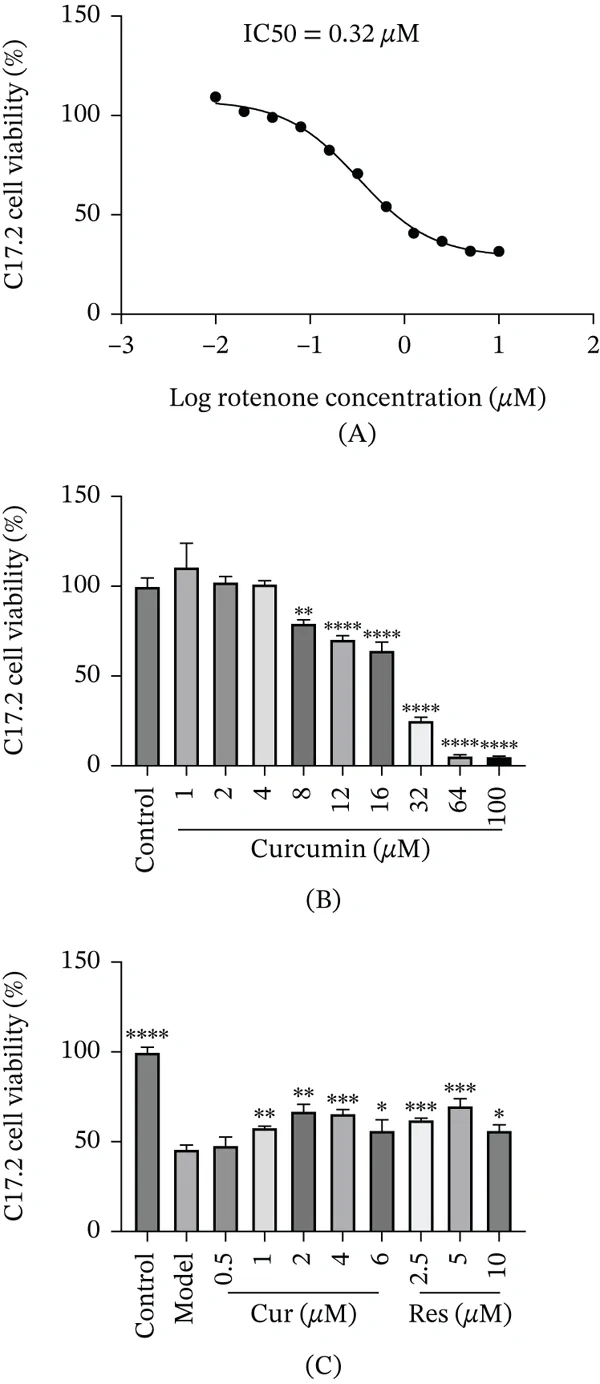
Cur′s safeguarding influence on C17.2 neural progenitor cells activated by Rot exposure. (A) Assessment of C17.2 cellular vitality post‐Rot induction (*n* = 3). (B) Cur′s effect on the viability of normal C17.2 cells. Findings are represented as the average ± standard deviation (*n* = 3). (C) Cur′s capacity to lessen Rot‐evoked harm in C17.2 cells. Results are depicted as mean ± SD (*n* = 3). In comparison to the model group,  ^∗^ *p* < 0.05,  ^∗∗^ *p* < 0.01,  ^∗∗∗^ *p* < 0.001,  ^∗∗∗∗^ *p* < 0.0001.

In the experiment to investigate the effect of Rot on the toxicity of C17.2 cells, the experimental data showed that the IC_50_ reached 0.32 *μ*mol/L (Figure 2A). When investigating the effect of Cur on C17.2 cells, the low concentration ranges (1, 2, 4 *μ*mol/L) of Cur showed almost no toxic effects on the cells, but the cell viability showed a significant decrease when the concentration climbed to 8 *μ*mol/L (Figure 2B). Based on the aforementioned findings, this study selected a lower concentration range of Cur (0.5, 1.0, 2.0, 4.0, and 6.0 *μ*mol/L) for subsequent experiments. Compared with the 100% survival benchmark of the control group, the cell survival rate of the model group alone plummeted to 45.85 ± 2.35%. Two.0 *μ*mol/L Cur showed the best cytoprotective effect, increasing the survival rate to 68.26 ± 1.97%. The 1.0 *μ*mol/L and 4.0 *μ*mol/L Cur‐treated groups followed suit, with survival rates of 65.67 ± 2.3% and 57.95 ± 0.68%, respectively. Five.0 *μ*mol/L Res also demonstrated a strong protective effect, with a high cell survival rate of 68.25 ± 3.72% (Figure 2C).

**Figure 3:**
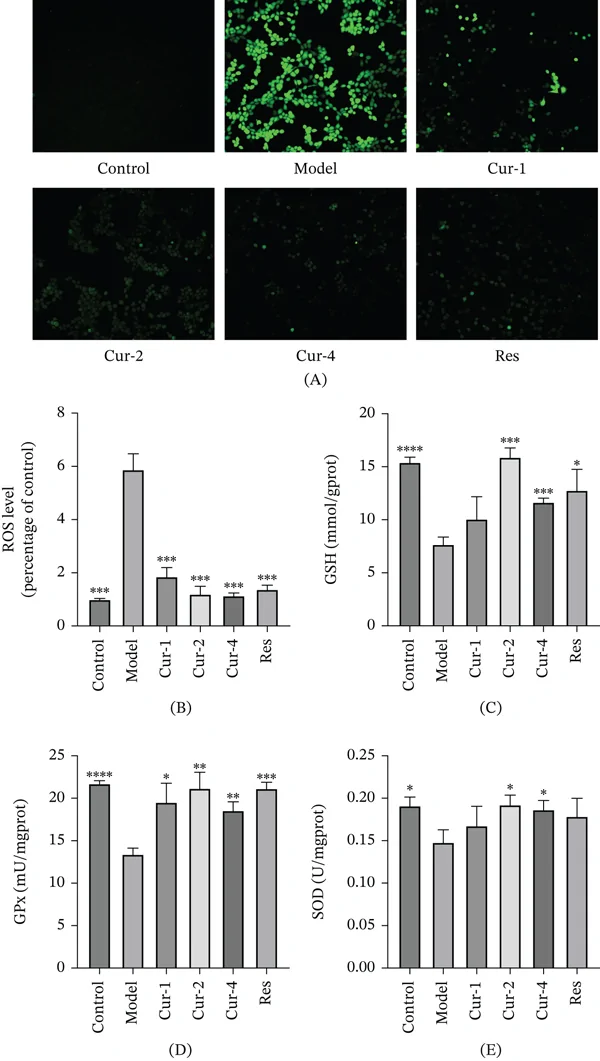
The protective effect of Cur on oxidative damage in C17.2 NSCs. (A) Fluorescence microscopy images at 100× magnification depict ROS levels in C17.2 cells post‐Rot treatment. (B) Cur′s role in modulating ROS fluorescence intensity within C17.2 cells is showcased (*n* = 3). (C) The effect of Cur on the GSH levels in C17.2 cells is detailed (*n* = 3). (D) The alterations in GPx activity in C17.2 cells due to Cur are highlighted (*n* = 3). (E) Cur′s sway on SOD activity in C17.2 cells is presented (*n* = 3). In comparison to the model group,  ^∗^ *p* < 0.05,  ^∗∗^ *p* < 0.01,  ^∗∗∗^ *p* < 0.001,  ^∗∗∗∗^ *p* < 0.0001.

In the experimental design stage, selected three gradient concentrations of Cur based on the data from previous studies: 1.0 *μ*mol/L (Cur‐1) in the low‐dose group, 2.0 *μ*mol/L (Cur‐2) in the medium‐dose group, and 4.0 *μ*mol/L (Cur‐4) in the high‐dose group, and 5.0 *μ*mol/L of Res as a positive reference. The experimental data showed that the fluorescence intensity of ROS in the model increased significantly with a relative expression of 5.88 ± 0.42, using the control ROS baseline as a reference, and strikingly, the ROS levels of both Cur‐intervened and Res showed a significant decrease (Figure 3A, B). The GSH level was maintained at 15.42 ± 0.34 mmol/gprot in the control, whereas it plummeted to 7.69 ± 0.48 mmol/gprot in the model. Figure 3C demonstrates the increase in GSH levels in the Cur‐2 and Cur‐4 as well as in the Res compared with the model. In terms of GPx activity, the control started at 21.73 ± 0.25 mU/mgprot, whereas the model dropped dramatically to 13.43 ± 0.58 mU/mgprot. All Cur concentration groups and the Res showed more active GPx activity than the model (Figure 3D). The total SOD activity was 0.18 ± 0.01 U/mgprot in the control and slightly decreased to 0.16 ± 0.01 U/mgprot in the model. From the data in Figure 3E, the Cur‐2 and Cur‐4 showed a significant advantage in total SOD activity.

Based on the significantly elevated GSH content and GPx activity observed in the Cur‐2 group compared with other Cur concentrations, this concentration was selected for subsequent experimental investigations.

### 3.3. Cur Promoted the Differentiation of C17.2 Cells Into Neurons and Astrocytes Following Oxidative Stress‐Induced Injury

**Figure 4:**
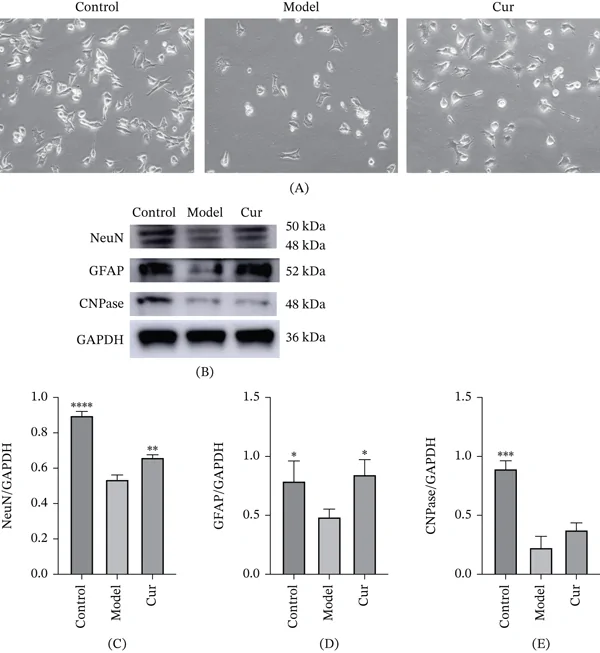
Cur facilitated the differentiation of C17.2 cells exposed to Rotenone. (A) Microscopic analysis at 200× magnification revealed distinct morphological changes in C17.2 cells across various groups. (B–E) WB was employed to evaluate the expression levels of NeuN, GFAP, and CNPase proteins (*n* = 3). In comparison to the model group,  ^∗^ *p* < 0.05,  ^∗∗^ *p* < 0.01,  ^∗∗∗^ *p* < 0.001,  ^∗∗∗∗^ *p* < 0.0001.

Following a 24 h differentiation period, the C17.2 cells in the control group showcased elongation. But when these cells were subjected to Rot stimulation, only a select few sprouted visible neuronal projections. In comparison, the cell bodies of the nondifferentiated C17.2 cells appeared smaller and misshapen. On the flip side, after exposure to a 2.0 *μ*mol/L concentration of Cur, a markedly greater number of C17.2 cells adopted a classic differentiated appearance, with longer neuronal processes and a more intricate network of branches, even establishing synaptic contacts, creating an interconnected network (Figure 4A).

WB results showed that the model displayed notably lower levels of NeuN, GFAP, and CNPase compared with the control. After Cur administration, there was a significant upsurge in NeuN and GFAP expressions, and although CNPase expression improved, it did not make the statistical significance (Figure 4B–E). Figure 4B shows the original blots, with GAPDH serving as the loading control (Figure S4)B.

**Figure 5:**
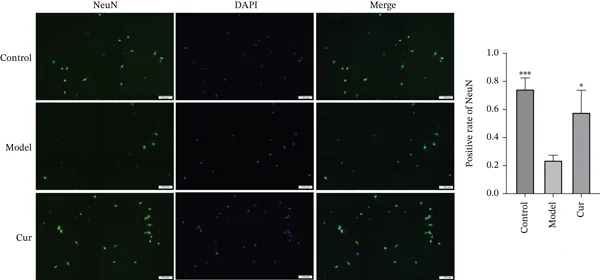
Immunofluorescence was employed to further detect the effect of Cur on neurogenesis (*n* = 3). In comparison to the model group,  ^∗^ *p* < 0.05,  ^∗∗∗^ *p* < 0.001.

IF assays further demonstrated that Cur can facilitate the differentiation of neuronal cells following Rot‐induced injury (Figure 5).

### 3.4. Cur Activated the Wnt3*α*/*β*‐Catenin/Cyclin D1 Pathway in the C17.2 PD Model

**Figure 6:**
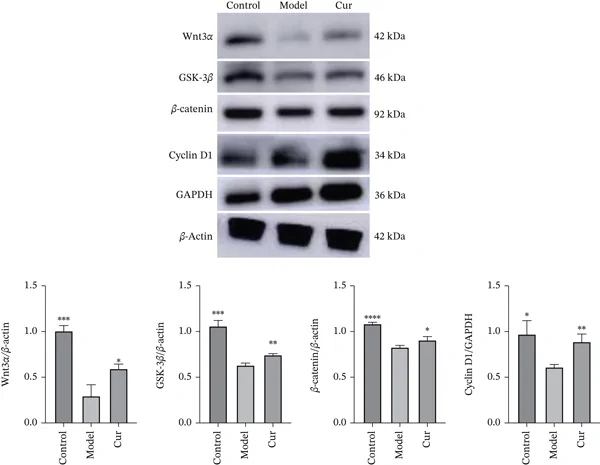
Cur facilitated the differentiation process of C17.2 neural stem cells post‐Rot injury by triggering the Wnt3*α*/*β*‐catenin/Cyclin D1 signaling cascade. WB was employed to gauge the protein concentrations of Wnt3*α*, GSK‐3*β*, *β*‐catenin, and Cyclin D1 (*n* = 3). In comparison to the model group,  ^∗^ *p* < 0.05,  ^∗∗^ *p* < 0.01,  ^∗∗∗^ *p* < 0.001,  ^∗∗∗∗^ *p* < 0.0001.

As illustrated in Figure 6, the protein expression levels of Wnt3*α*, GSK‐3*β*, *β*‐catenin, and Cyclin D1 were markedly reduced in the model compared with the control. In contrast, Cur treatment significantly upregulated the expression levels of these proteins. These findings suggest that Cur enhances the differentiation of C17.2 NSCs following Rot‐induced injury by activating the Wnt3*α*/*β*‐catenin/Cyclin D1 signaling pathway. Figure 6 shows the original blots, with *β*‐actin serving as the loading control. (Figure 6).

## 4. Discussion

PD is characterized by the prominent pathological feature of degeneration and death of DA neurons in the substantia nigra. Current primary pharmacological treatments for PD primarily target the dopamine system. These include levodopa‐carbidopa, which provides exogenous dopamine replacement, and agents like amantadine, which enhance endogenous dopamine release [12]. However, these traditional therapies often cause troubling side effects during long‐term application and may even lead to a state of “off” in which the therapeutic effect plummets and the symptoms rebound [13]. Against this background, stem cell therapy opens up new possibilities for treatment through both transplantation and stimulation of endogenous stem cell differentiation [14]. NSCs are notable for their potential to differentiate into neurons, astrocytes, and oligodendrocytes, a multidirectional differentiation capability that provides an ideal cellular source for targeted therapies [15]. These cells are naturally present in the subventricular zone surrounding the striatum, and through appropriate activation mechanisms, they are able to migrate to the striatum and achieve neuronal transformation, thereby promoting neurological self‐repair and offering new therapeutic hope for PD patients [16]. Cur, a potent antioxidant derived from turmeric, exhibits neuroprotective effects that may alleviate PD by mitigating oxidative stress, ameliorating mitochondrial dysfunction, and reducing *α*‐synuclein aggregation [17]. Besides, numerous scholarly investigations have shown that Cur can spur the differentiation of NSCs by switching on the Wnt3*α*/*β*‐catenin signaling pathway [18]. Study revealed that Cur promotes the differentiation of activated endogenous NSCs into neurons, and consequently enhances the recovery of hindlimb function in rats with spinal cord injury by activating the Wnt3*α*/*β*‐catenin signaling pathway [19].

C17.2 NSCs spontaneously acquire DA traits upon striatal transplantation, making them a validated model for PD research [20].

The expression level of the intermediate filament protein Nestin, an important marker of neuroepithelial stem cell characteristics, directly reflects the stemness of the cells [21]. Accompanying the differentiation process, the expression of three specific proteins, GFAP, NeuN, and CNPase, marked the formation of astrocytes, neurons, and oligodendrocytes, respectively [22]. In this study, the biological characteristics of the C17.2 NSC line were evaluated through IF combined with morphological observation. The results demonstrated that this cell line sustained stable expression of Nestin protein while retaining differentiation potential. These findings not only confirm that C17.2 cells maintain typical NSC properties but also possess the capacity for differentiation, thus providing a valuable in vitro model for subsequent research.

To model the pathological state of PD neurons, Rot was employed to establish an in vitro cell model. Previous experimental results indicated that Rot significantly reduced the viability of C17.2 cells and elevated intracellular ROS levels, while concurrently decreasing GSH content, GPx activity, and SOD activity, thereby inducing an oxidative stress state. Treatment with Cur within a specific concentration range ameliorated the Rot‐induced decline in C17.2 cell survival, significantly enhancing cellular viability. Cur also effectively reduced ROS accumulation and increased GSH levels, GPx, and SOD activities. Among the concentrations tested, 2.0 *μ*mol/L Cur demonstrated the most potent protective effect and was consequently selected for use in subsequent experiments. Morphological assessment revealed that Cur‐treated C17.2 cells exhibited improved overall condition and displayed characteristic differentiation morphology, including longer neurites, more complex branching, and the formation of synaptic connections. In contrast, cells in the Rot model group predominantly exhibited rounded and atrophic morphologies. Furthermore, WB analysis demonstrated that Rot‐induced injury significantly downregulated the expression levels of neuronal markers NeuN, astrocytic marker GFAP, and oligodendrocytic marker CNPase in the model group. Notably, treatment with 2.0 *μ*mol/L Cur significantly upregulated NeuN and GFAP expression. Although an increase in CNPase expression was observed, it did not reach statistical significance. These findings collectively indicate that Cur promotes the differentiation of C17.2 cells into neurons and astrocytes under Rot‐induced oxidative stress conditions. This neurogenic effect was further corroborated by IF results. Having established that Cur alleviates oxidative stress and promotes differentiation in NSCs, we subsequently investigated the involvement of the Wnt3*α*/*β*‐catenin pathway using WB. The results showed that Rot markedly suppressed the protein expression levels of Wnt3*α*, GSK‐3*β*, *β*‐catenin, and Cyclin D1 in C17.2 cells. Conversely, Cur treatment partially restored the expression of these proteins. Taken together, these results suggest that Cur stimulates the differentiation of Rot‐injured C17.2 NSCs, at least in part, by activating the Wnt3*α*/*β*‐catenin/Cyclin D1 signaling pathway.

We found that Cur appears to preferentially promote neuronal/astrocytic over oligodendrocytic differentiation. Several explanations may account for our observations. Firstly, pathway specificity is a critical factor: Cur inhibits GSK‐3*β*, thereby activating Wnt/*β*‐catenin signaling, which predominantly promotes neurogenesis over oligodendrogenesis in NSCs, as the Wnt pathway is more strongly associated with neuronal fate specification. Secondly, differential sensitivity is noteworthy: lineage commitments likely exhibit distinct dose–response thresholds [23]. For example, a concentration of 10 *μ*m Cur inhibits astrocytic and immature neuronal differentiation, whereas lower concentrations (0.5–5 *μ*m) facilitate neuronal differentiation. This suggests that oligodendrocyte differentiation may necessitate alternative cues or higher concentrations. Thirdly, the biological significance in the context of PD is evident. PD is primarily characterized by the loss of DA neurons rather than oligodendrocyte pathology. Therefore, the preferential promotion of neuronal (TH‐positive) differentiation is therapeutically advantageous, as NSC‐based therapies aim to replenish lost neurons. Furthermore, astrocytic differentiation may offer trophic support for newly formed neurons. Recent research underscores Cur′s propensity to promote neurogenesis, as evidenced by a study examining its effects on the proliferation and neurogenic differentiation of embryonic NSCs [24]. However, Cur has also been shown to facilitate oligodendrocyte differentiation through the activation of PPAR‐*γ*. These findings suggest that experimental variables, including cell source, culture medium, concentration, and exposure duration, play a pivotal role in determining lineage‐specific outcomes.

Wnt3*α*, a highly representative signaling protein within the Wnt family, is widely distributed in biological systems. It activates the canonical Wnt signaling pathway by specifically binding to Frizzled receptors on the cell membrane, initiating downstream signaling cascades. This pathway plays a crucial role in regulating pleiotropic cellular functions, particularly the development and differentiation of the nervous system [25]. GSK‐3*β*, a key serine/threonine kinase, plays a pivotal role in cellular signal transduction and metabolic regulation. *β*‐catenin is a multifunctional protein involved not only in the assembly and maintenance of the cytoskeleton but also in interacting with extracellular signaling molecules via its unique domains. Crucially, *β*‐catenin acts as a transcriptional coactivator within the nucleus, directly regulating gene expression to promote cell proliferation and differentiation. Within the homeostatic regulation of the canonical Wnt pathway, GSK‐3*β* serves as a key component of the *β*‐catenin destruction complex. This complex binds to the N‐terminal phosphorylation sites of *β*‐catenin in a spatially specific conformation, enabling GSK‐3*β* to sequentially phosphorylate *β*‐catenin. This phosphorylation tags *β*‐catenin for degradation via the ubiquitin‐proteasome pathway [26].

Sahebdel et al. demonstrated that activation of the Wnt/*β*‐catenin pathway upregulates its downstream targets c‐Myc and Cyclin D1, concomitant with increased expression of DA markers Nurr‐1 and MAP2, thereby driving cells toward a DA fate [27].

Our finding that Wnt3*α* inhibition coincided with reduced GSK‐3*β* expression may be explained by two nonmutually exclusive possibilities: the temporal dynamics at the selected time points, or a functional crosstalk between the Wnt cascade and the PI3K/Akt pathway—the latter being particularly relevant under oxidative stress and capable of promoting GSK‐3*β* degradation. In support of this, Almeida et al. (2005) demonstrated that Wnt3a‐induced phosphorylation of GSK‐3*β* and subsequent *β*‐catenin‐mediated transcription require ERK, PI3K, and Akt signaling, underscoring the interconnected regulation of these pathways [28].

As a key node in the cell cycle regulatory network, Cyclin D1 not only promotes the cell proliferation process, but also plays a unique role in neuronal differentiation [29]; in depth, Cyclin D1 promotes the G1/S phase transition by forming a complex with CDK4/6 while possessing the function of regulating neurogenesis [30]. Collectively, the Wnt3*α*/*β*‐catenin/Cyclin D1 signaling pathway exerts critical regulatory functions in neurogenesis. This is achieved through the precise control of *β*‐catenin stability, which subsequently modulates the expression and activity of Cyclin D1.

This study has several limitations that warrant acknowledgment. Firstly, as this research serves as the experimental validation component of a larger project, the upstream network pharmacology data were generated by external groups, which may introduce potential bias or heterogeneity. Secondly, although the differentiation protocol has been previously validated in other studies, we did not conduct cross‐laboratory reproducibility tests. We acknowledge that the present study did not include loss‐of‐function rescue experiments (e.g., using Wnt pathway inhibitors such as IWR‐1 or DKK1, or *β*‐catenin siRNA) to directly establish causality. Although multiple independent studies have demonstrated that pharmacological blockade of the Wnt/*β*‐catenin pathway abolishes Cur′s neurogenic effects, a limitation of our study is that we did not perform such experiments in our system. Thirdly, our temporal analysis was primarily concentrated on the 24‐h time point, with only limited verification at 48 and 72 h; consequently, later events such as neuronal maturation and functional integration were not captured. Lastly, the absence of an animal model limits our ability to assess the in vivo efficacy of Cur. As a result, our findings are predominantly mechanistic and cell‐autonomous. Future research should incorporate multiple time points, diverse cell lines, and appropriate animal models (e.g., MPTP‐ or 6‐OHDA‐treated mice) to validate and expand upon these observations.

## 5. Conclusion

Cur significantly enhanced the viability of C17.2 NSCs under Rot‐induced oxidative stress. This protective effect was characterized by reduced intracellular ROS levels, elevated GSH content, and restored activities of GPx and SOD, collectively ameliorating cellular oxidative stress. Furthermore, Cur markedly potentiated the differentiation capacity of oxidatively damaged C17.2 NSCs into neurons and astrocytes. Mechanistically, our findings suggest that Cur likely facilitates NSC differentiation by activating the Wnt3*α*/*β*‐catenin/Cyclin D1 signaling pathway.

This study is limited to in vitro experiments with cultured NSCs, lacking animal studies. Thus, the results mainly reveal cellular mechanisms, not in vivo efficacy. Future research will use toxin‐induced PD models to assess Cur′s effects on neurogenesis, neuron protection, and the Wnt3*α*/*β*‐catenin pathway in vivo. Animal studies are crucial to confirm Cur′s potential for clinical use.

**Nomenclature**
CNPase: 2 ^′^,3 ^′^‐cyclic adenylate 3 ^′^‐phosphodiesterase
Cur: curcumin
DA: dopaminergic
GFAP: glial fibrillary acidic protein
GPx: glutathione Peroxidase
GSH: glutathione
NeuN: neuron‐specific nuclear protein
NSCs: neural stem cells
PBS: phosphate‐buffered saline
PD: Parkinson′s disease
Res: resveratrol
ROS: reactive oxygen species
Rot: rotenone
SOD: superoxide dismutase
WB: Western blot

## Funding

China Academy of Chinese Medical Sciences Medical Experimental Research Center, CI2026A05008.

## Conflicts of Interest

There are no conflicts of interest in this manuscript.

## Supporting Information

Additional supporting information can be found online in the Supporting Information section.

## Supporting information


**Supporting Information 1** Figure S4B shows the original Western blot bands for differentiation markers (NeuN, GFAP, and CNPase) in C17.2 cells.

**Supporting Information 2** Figure S6 shows the original bands for Wnt3*α*, GSK‐3*β*, *β*‐catenin, and Cyclin D1, with *β*‐actin serving as the loading control for both figures.

## Data Availability

The data that support the findings of this study are available from UF Health . Restrictions apply to the availability of these data, which were used under license for this study. Data are available from https://idr.ufhealth.org/research‐services/research‐data/ with the permission of UF Health.
