# Supplementary material for: Experimental Study on the Effects of Curcumin in Alleviating Oxidative Stress and Inducing Differentiation in Neural Stem Cells
Source: Int J Food Sci. 2026 Sep 20;2026:7647912. doi: 10.1155/ijfo/7647912 (PMC13591120; doi:10.1155/ijfo/7647912)

CNPase:

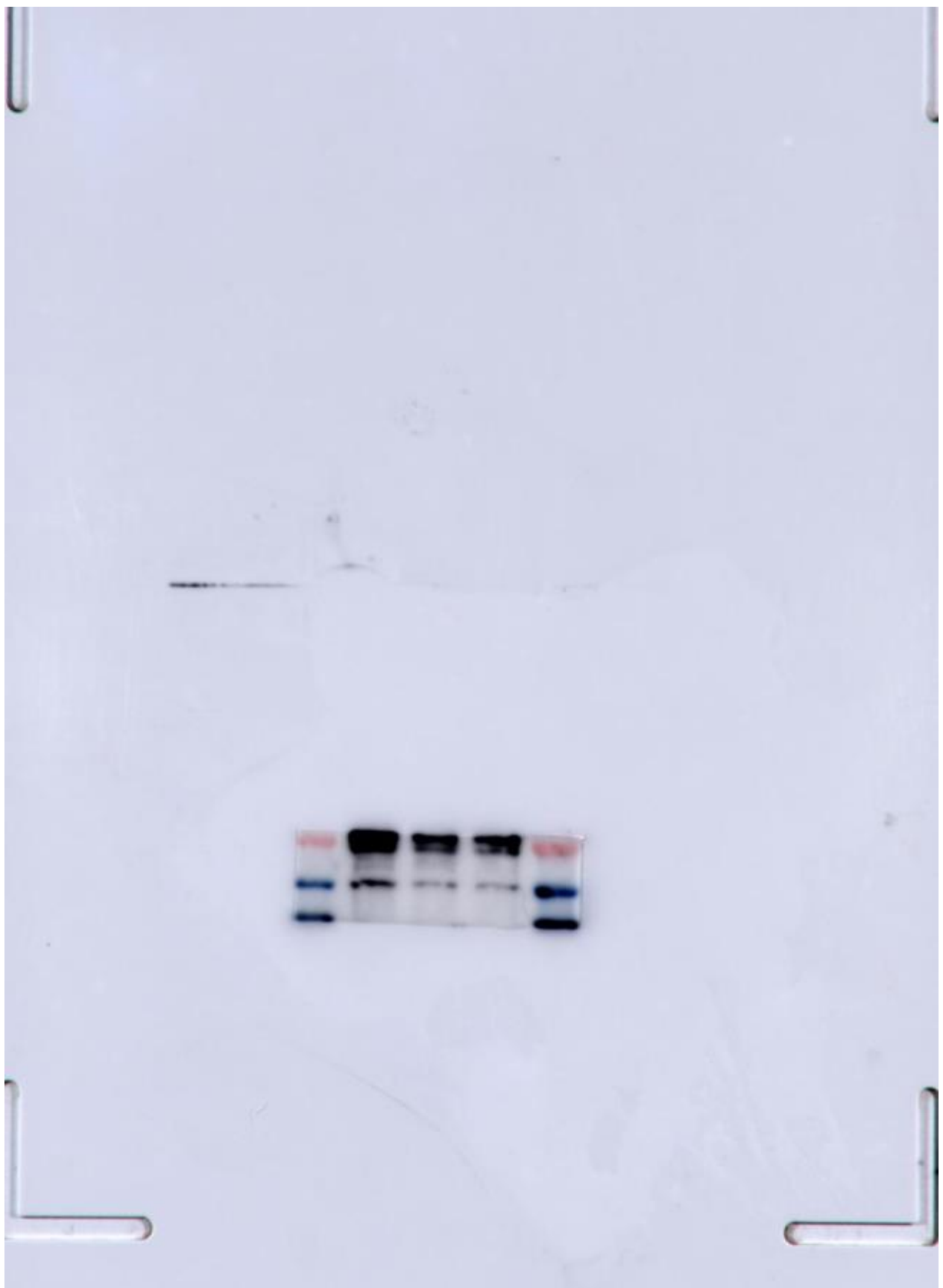

GAPDH:

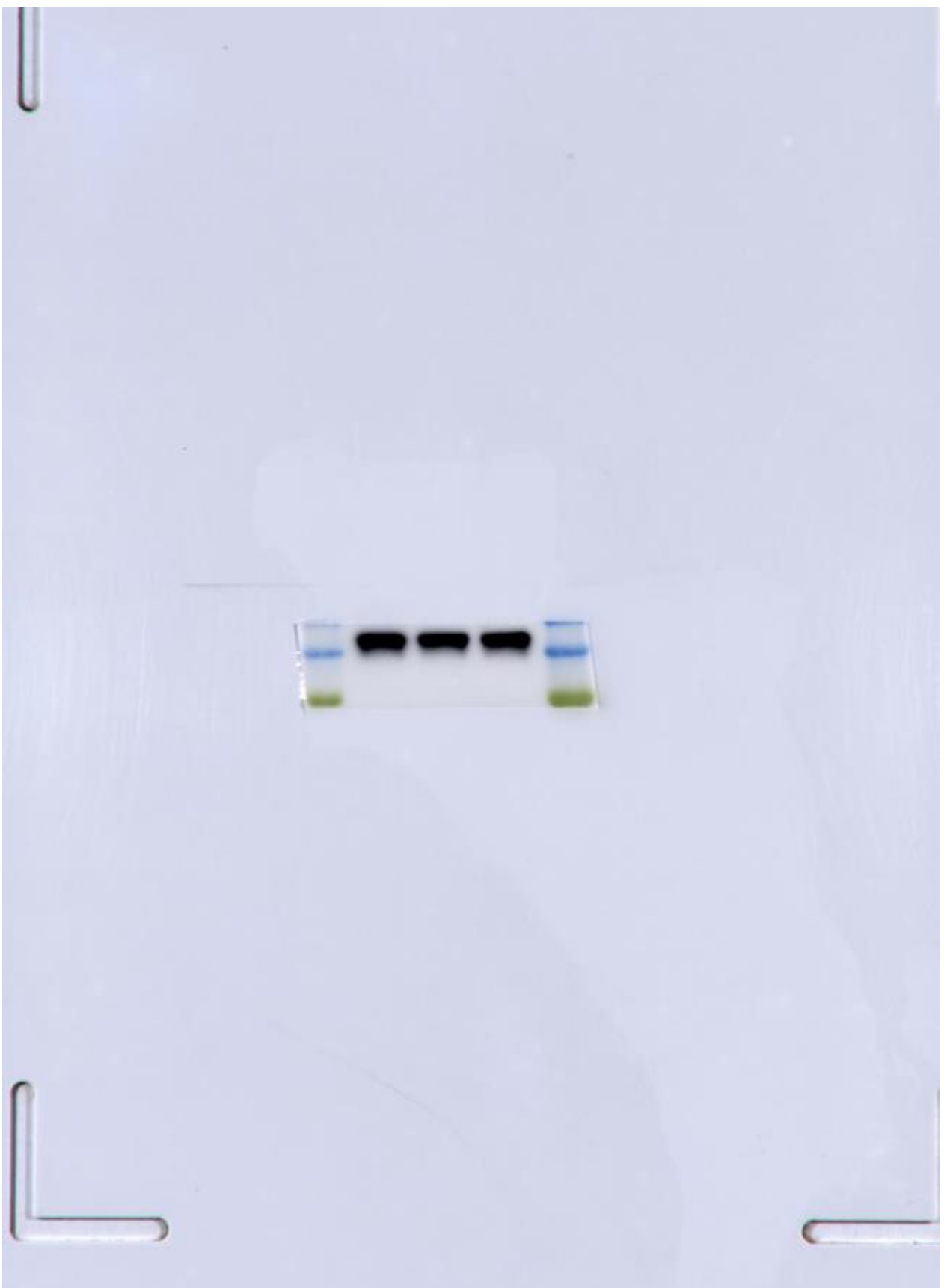

GFAP:

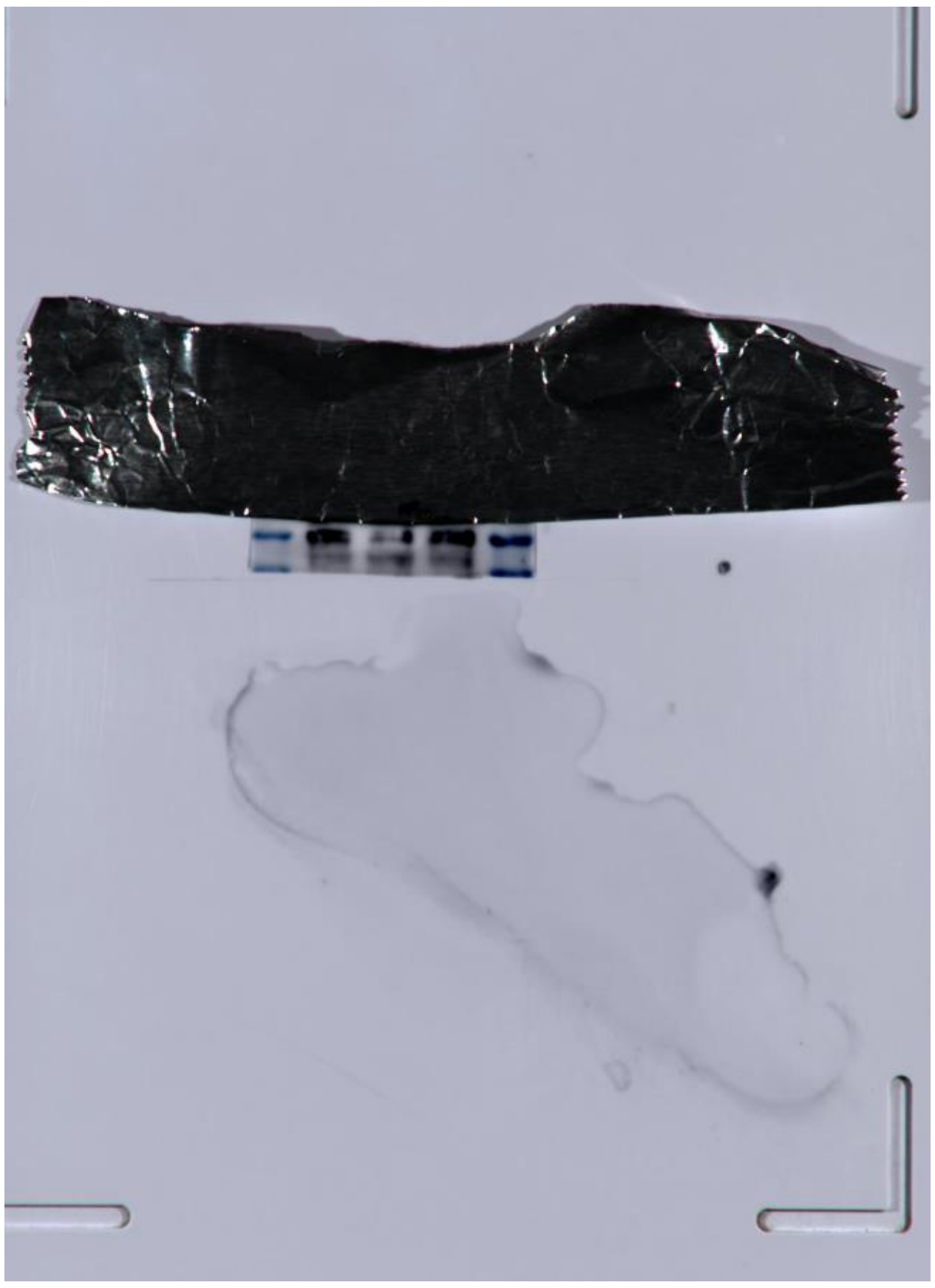

NeuN(The three rightmost lanes are circled in red.):

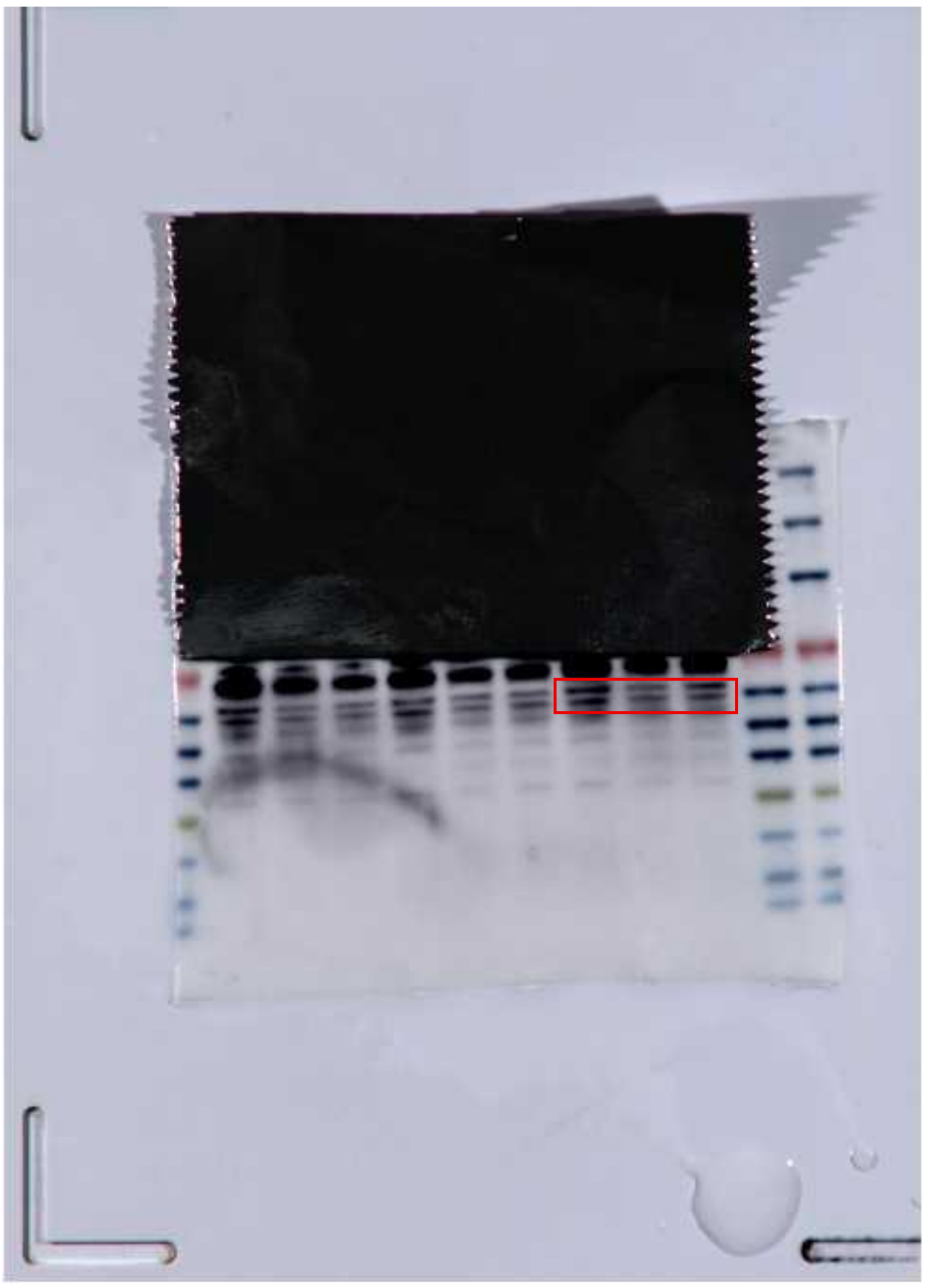

Supplement: Supplementary file 1 [file IJFO-2026-7647912-s001.pdf]
