# Supplementary material for: Experimental Study on the Effects of Curcumin in Alleviating Oxidative Stress and Inducing Differentiation in Neural Stem Cells
Source: Int J Food Sci. 2026 Sep 20;2026:7647912. doi: 10.1155/ijfo/7647912 (PMC13591120; doi:10.1155/ijfo/7647912)

β-actin(The three middle lanes are circled in red):

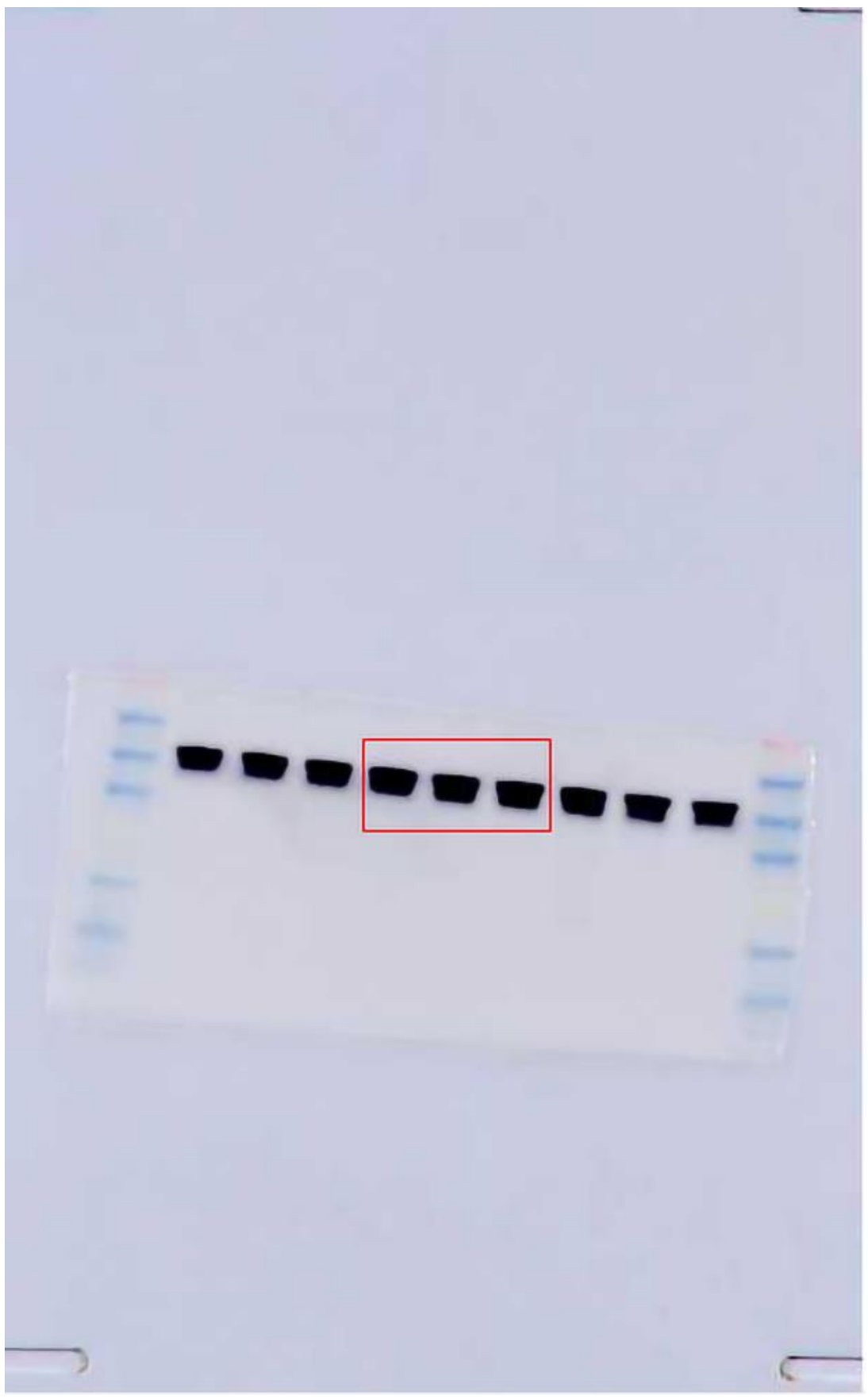

β-catenin (The three middle lanes are circled in red):

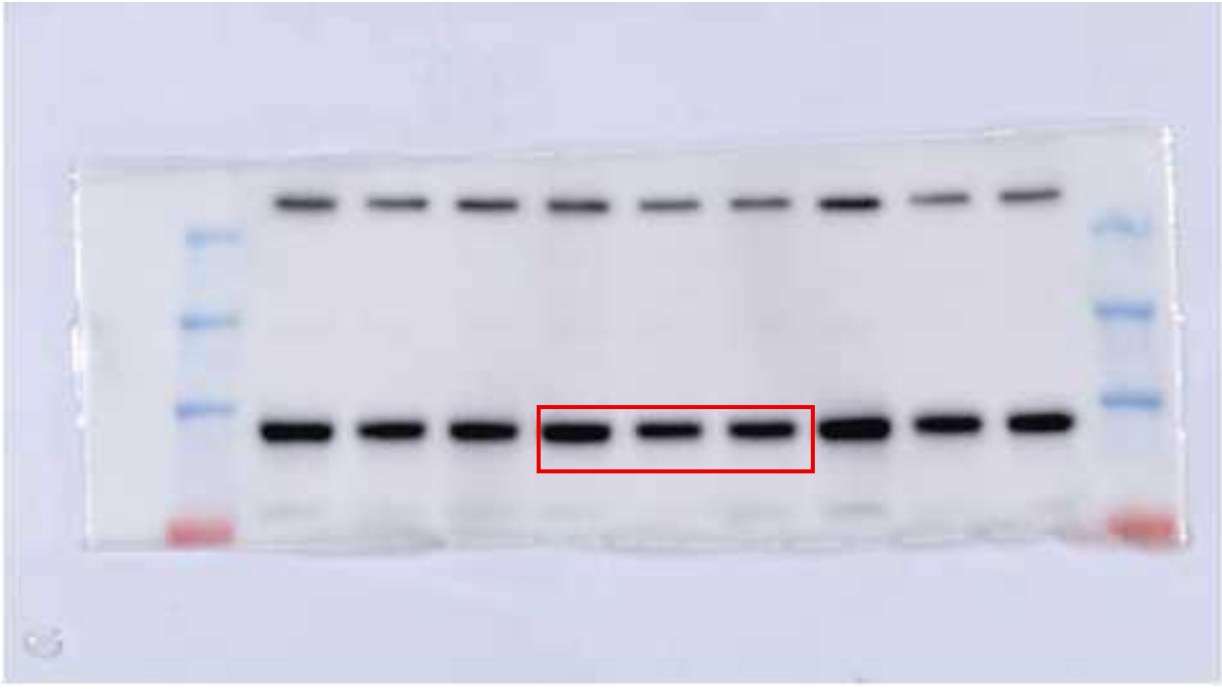

Cyclin D1:

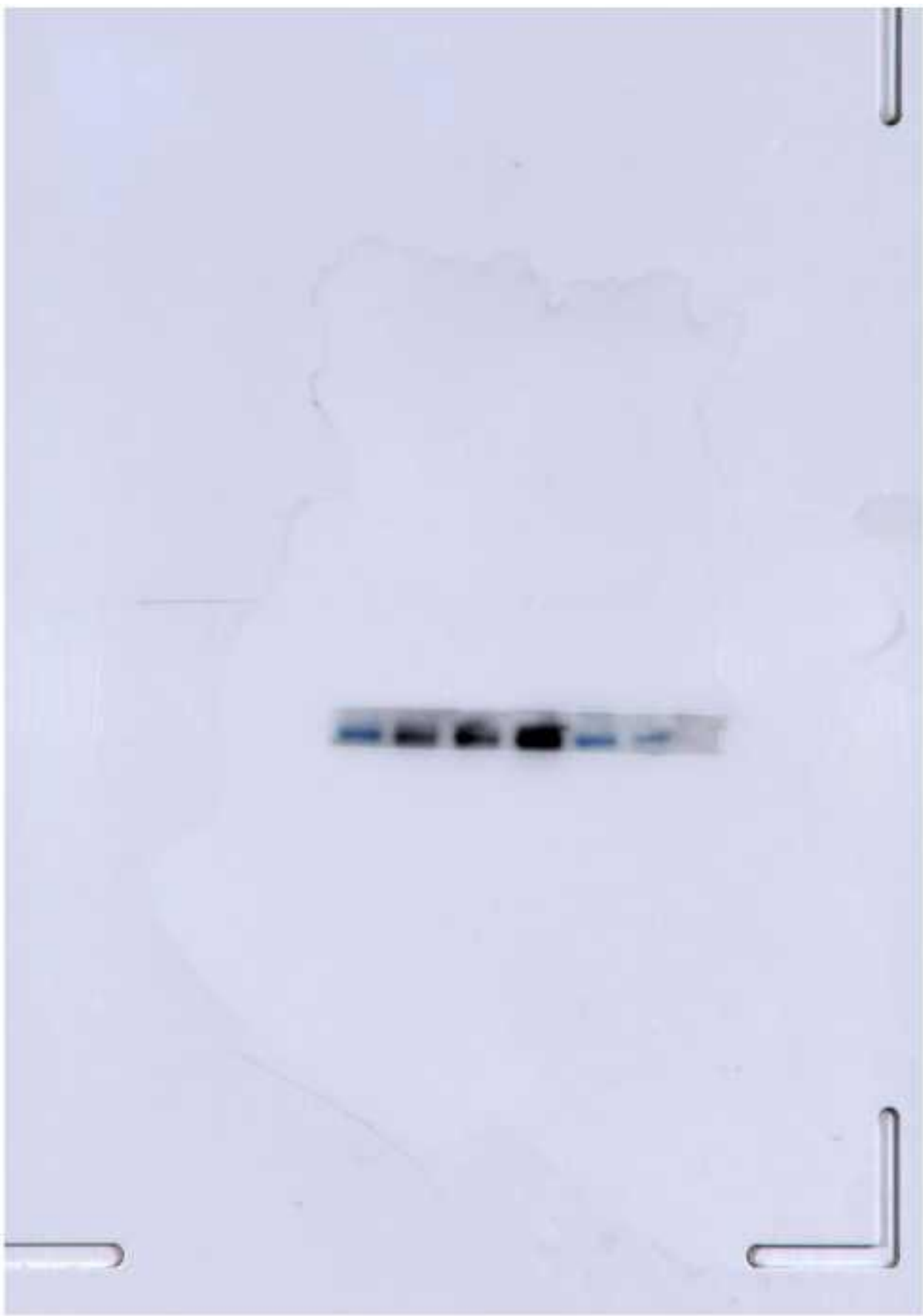

GAPDH:

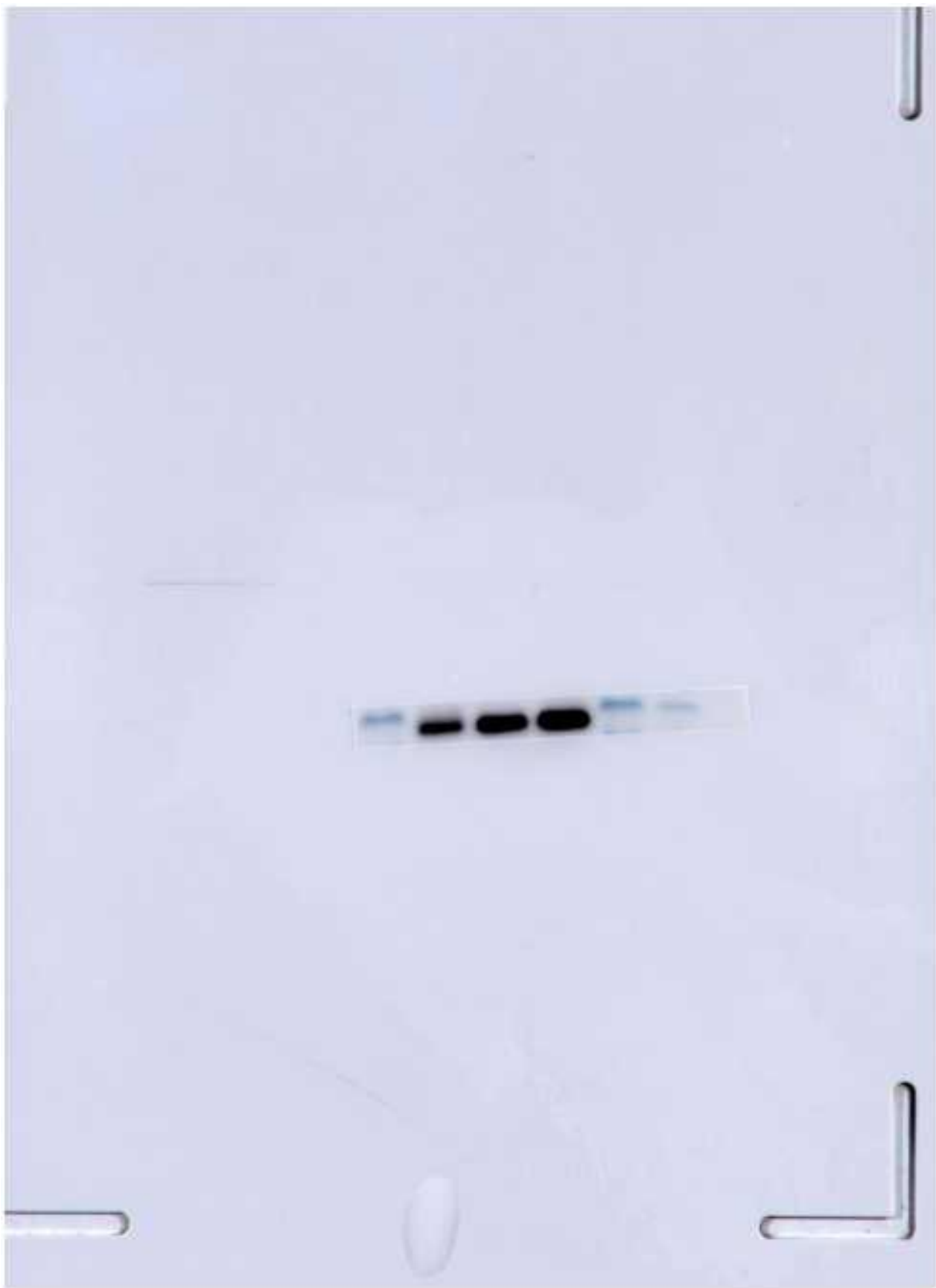

GSK-3β(The three rightmost lanes are circled in red):

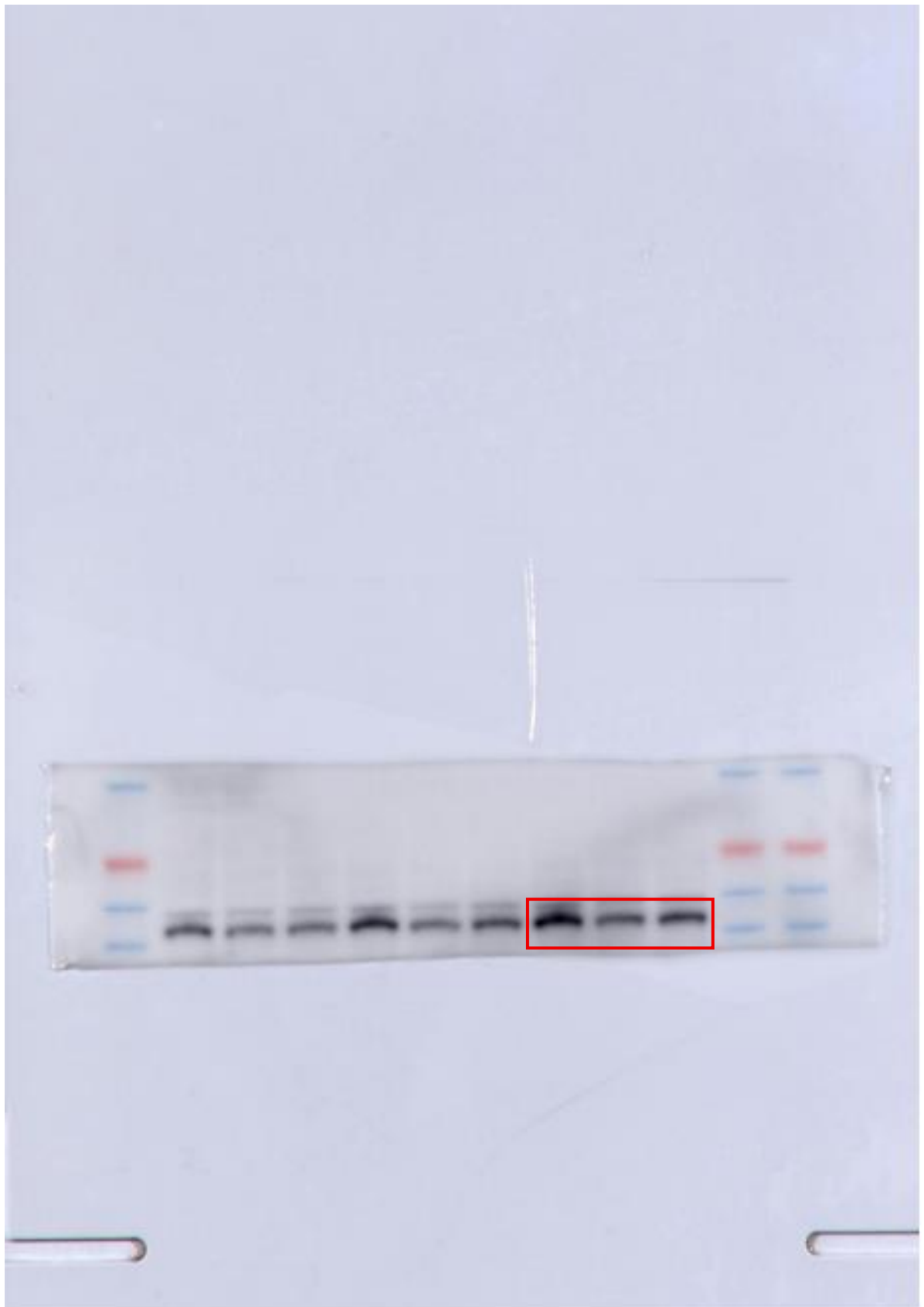

Wnt3α(The three middle lanes are circled in red):

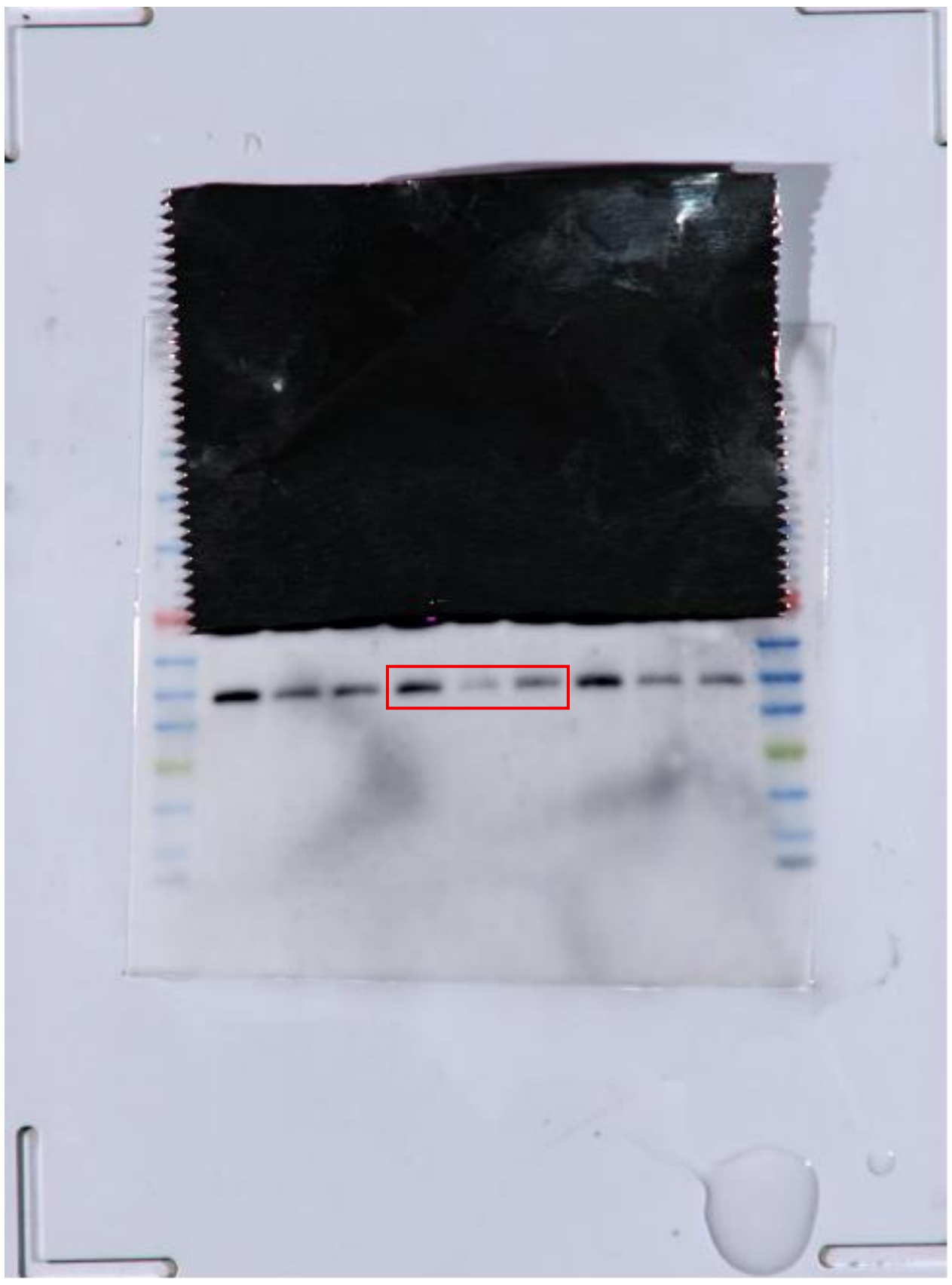

Supplement: Supplementary file 2 [file IJFO-2026-7647912-s002.pdf]
